# On the adhesion-cohesion balance and oxygen consumption characteristics of liver organoids

**DOI:** 10.1371/journal.pone.0173206

**Published:** 2017-03-07

**Authors:** Giorgio Mattei, Chiara Magliaro, Serena Giusti, Sarada Devi Ramachandran, Stefan Heinz, Joris Braspenning, Arti Ahluwalia

**Affiliations:** 1 Research Centre "E. Piaggio", University of Pisa, Largo Lucio Lazzarino, Pisa, Italy; 2 Medicyte GmbH, Im Neuenheimer Feld, Heidelberg, Germany; 3 Tissue Engineering and Regenerative Medicine, University Hospital Wuerzburg, Roentgering, Wuerzburg, Germany; Technische Universitat Dresden, GERMANY

## Abstract

Liver organoids (LOs) are of interest in tissue replacement, hepatotoxicity and pathophysiological studies. However, it is still unclear what triggers LO self-assembly and what the optimal environment is for their culture. Hypothesizing that LO formation occurs as a result of a fine balance between cell-substrate adhesion and cell-cell cohesion, we used 3 cell types (hepatocytes, liver sinusoidal endothelial cells and mesenchymal stem cells) to investigate LO self-assembly on different substrates keeping the culture parameters (e.g. culture media, cell types/number) and substrate stiffness constant. As cellular spheroids may suffer from oxygen depletion in the core, we also sought to identify the optimal culture conditions for LOs in order to guarantee an adequate supply of oxygen during proliferation and differentiation. The oxygen consumption characteristics of LOs were measured using an O_2_ sensor and used to model the O_2_ concentration gradient in the organoids. We show that no LO formation occurs on highly adhesive hepatic extra-cellular matrix-based substrates, suggesting that cellular aggregation requires an optimal trade-off between the adhesiveness of a substrate and the cohesive forces between cells and that this balance is modulated by substrate mechanics. Thus, in addition to substrate stiffness, physicochemical properties, which are also critical for cell adhesion, play a role in LO self-assembly.

## Introduction

The growing evidence that three-dimensional (3D) microenvironments contribute critically to tissue function has led to the rapid development of cellular organoids. That cells aggregate spontaneously *in vitro* has been known for decades, however only recently have scientists begun to manipulate stem cells and different parenchymal cell types in different conditions to generate mini-functional organs. We now know that in the right conditions (cell number, cell types, substrate, agitation) and with the right timing (addition of differentiating media), stem cells proliferate and self-organize to form tissue proxies known as organoids [1–3]. Since they recapitulate the *in vivo* micro-environment to a large degree, self-assembled tissue organoids can be used to study development, toxicity and diseases or can be applied to tissue engineering and drug development. However, although the key players in organoid formation have been identified, it is still unclear what triggers cellular self-assembly and what the optimal environment is for culturing and maintaining these mini-organs.

Given the growing incidence of chronic liver disease as well as the organ’s importance in drug metabolism, liver organoids (LOs) are of particular interest for their wide range of potential applications in both medicine and in the pharmaceutical industry. Takebe and co-workers first described the generation of liver organoids, using a combination of human hepatocyte-like cells derived from induced pluripotent stem cells, endothelial cells and mesenchymal stem cells (MSCs) seeded onto substrates made of Matrigel^TM^ diluted in an equal volume of endothelial growth medium (i.e. x2 dilution) [2]. Increasing the Matrigel dilution negatively affected LO formation, which was almost absent at 8x and 16x dilution, while changing the substrate composition to only adhesive proteins (Laminin and Entactin, Laminin, Collagen I), or biochemically inert 1.5% agarose, completely inhibited LO formation. In a more recent study, the same group investigated the formation of LOs on polyacrylamide substrates with different stiffness, coated with Matrigel^TM^ diluted to 227x in HEPES [4]. Ramachandran et al. used the same principles to assemble LOs derived from adult human hepatocytes, liver sinusoid endothelial cells (LSECs) and MSCs, on Matrigel with an equal volume of endothelial growth medium (i.e. Matrigel x2) [5]. They observed that the organoids developed a necrotic core after a few days in static culture, indicating nutrient depletion in the centre of the bud.

Generating LOs for *in vitro* applications, such as assessment of drug safety and efficacy or disease models, requires that liver bud formation be reproducible and rapid and that the organoids maintain their functional capacity for several days to allow chronic testing. Therefore, in this study we addressed a number of questions pertinent to the optimization of LO formation and their culture *in vitro*: i) Is LO formation a matter of substrate properties, adjuncts such as growth factors, or both of them? ii) Is stiffness or adhesiveness the trigger for LO assembly? iii) What are the optimal conditions for LO culture to ensure adequate oxygen supply through to the core?

To identify the key players in LO formation, providing insights into the balance between substrate adhesion and cell cohesion, we first focused on substrate properties and tested LO formation on 8 different substrates keeping the culture parameters (e.g. culture media, cell types and numbers, etc.) constant among experiments. Specifically, we studied i) non-adhesive agarose substrates, ii) highly adhesive substrates derived from decellularised liver and iii) Matrigel. In some cases, to isolate effects due to adjunct growth factors, the materials were also investigated with and without the addition of endothelial cell growth medium (LSEC medium). Cells seeded onto Matrigel-LSEC medium substrates as described in Ramachandran et al. [5] were used as positive control for LO formation. We then set up an experimental protocol to measure LO oxygen consumption, correlating our results with those of LO glucose uptake. The results were used to predict the oxygen concentration gradient in the organoids and thus propose appropriate culture conditions to ensure their long-term viability *in vitro*. Finally, the adhesion-cohesion balance on different substrates was evaluated by quantifying the expression of integrin-β1 and connexin-32.

## Materials and methods

### Cell source

Human upcyte® hepatocytes, upcyte® LSECs and upcyte® MSCs, upcyte® Hepatocyte Growth Medium and upcyte® High Performance Medium, upcyte® LSEC Medium were obtained from Medicyte GmbH (Heidelberg, Germany). Foetal bovine serum was obtained from PAN GmbH (Aidenbach, Germany). The upcyte process was performed according to Burkard et al. [6]. Cells were cultured in a humidified incubator (37°C, 5% CO_2_, 95% humidity) and passaged at 70–80% confluence. Living cells were counted using Trypan Blue exclusion.

### Cell culture substrates

#### Matrigel^TM^ hydrogels

BD Matrigel™ Basement Membrane Matrix was obtained from Corning. The gel was thawed overnight at 4°C, then diluted 1:1 with upcyte® LSEC Growth Medium, obtaining the Matrigel-LSEC precursor solution for positive control experiments, as described in Ramachandran et al. [5].

Since the upcyte® LSEC Growth Medium used for preparing Matrigel-LSEC substrates contains adjuncts including foetal bovine serum (FBS) and growth factors (GFs) which may affect LO formation, Matrigel^TM^ diluted 1:1 with PBS (named Matrigel-PBS) was also investigated as a substrate for LO formation to help in identifying the key player(s) triggering this spontaneous process.

For the generation of one liver organoid in a 24-well format, 380 μL of Matrigel-LSEC or Matrigel-PBS solution (~200 μL/cm^2^) were added to the plates/chambers and incubated at 37°C for 30 to 45 minutes to allow for polymerization. The culture plates and pipette tips were pre-cooled at -20°C before use to prevent Matrigel^TM^ gelation during preparation. The mechanical properties of Matrigel-PBS were assumed to be equal to those of Matrigel-LSEC. The compressive modulus of x2 diluted Matrigel substrates was estimated from literature as ~ 300 Pa [4–7].

#### Agarose gels

Agarose gels were prepared at different final concentrations (i.e. 0.01, 0.05, 0.1, 0.25, 0.5 and 1.5% w/v) in order to find the best one matching the stiffness of Matrigel x2-based substrates. This strategy allows decoupling the role of mechanical properties from other Matrigel-related signals in directing LO formation [7]. Agarose gels were prepared dissolving agarose powder (A9539, Sigma-Aldrich) in boiling deionised water (1/2 of final solution volume). The solution was then cooled to 40°C and LSEC medium added to reach the final volume. Then, 380 μL/well of the agarose-LSEC solution was cast in 24 well plates and cooled at 4°C for 1h to allow for gelation.

#### Liver-ECM gels

Substrates for liver organoid formation were also obtained from decellularised porcine liver extracellular matrix (dECM). Briefly, cylindrical liver samples were obtained from 1 year old healthy pigs as a slaughter by-product and decellularised using a 3 day long immersion and agitation procedure based on non-ionic detergents, which was shown to preserve key adhesive ECM proteins [8]. The liver dECMs obtained were lyophilised and ground into powder, then enzymatically digested using a 4 mg/mL pepsin solution (Pepsin from porcine gastric mucosa, P7012, Sigma-Aldrich) in 0.1 M HCl under moderate stirring for 48 h at room temperature, obtaining a 40 mg/mL liver ECM digest as reported by Lee et al. [9]. The digest was neutralised at pH 7.4 adding 0.5 M NaOH dropwise, then diluted with deionised water obtaining a 20 mg/mL ECM pre-gel solution, which was biochemically characterised in terms of total amino content (TAC) and total collagen content (TCC). The ECM pre-gel solution was aliquoted and stored at -20°C until use. As for Matrigel, the ECM pre-gel solution was thawed overnight at 4°C, then 380 μL of solution were added to 24 well plates and incubated at 37°C for 30 to 45 minutes to allow for polymerization. Diluted 10 mg/mL ECM-LSEC hydrogels were also investigated as substrate for LO formation by adding an equal volume of upcyte® LSEC Growth Medium to the 20 mg/mL ECM pre-gel solution.

### Liver ECM pre-gel solution biochemical characterisation

The Total Collagen Content (TCC) was determined using Sirius Red. A TCC calibration curve was made using standard samples of collagen type I from rat tail (C3867, Sigma-Aldrich) in 0.02 N CH_3_COOH. The Total Amino Content (TAC) was determined spectrophotometrically using the Ninhydrin assay. The TAC calibration curve was established using standard samples of cysteamine (Cysteamine ≥ 98.0% (RT), 30070, Sigma-Aldrich) prepared in PBS 1X and buffered to pH 7.4 (i.e. the same as the ECM pre-gel solution) to avoid any pH related bias. For both assays, a pepsin solution with no liver dECM powder was prepared (as described in ‘Liver ECM gels’) and used as a blank for absorbance readings of liver ECM pre-gel solution samples.

### Mechanical characterisation of agarose and liver ECM gels

Mechanical tests were performed on cylindrical samples of both agarose and ECM gels prepared in a custom designed mold with 13 mm diameter– 8 mm height wells. Unconfined compressive tests were performed at room temperature with a Zwick/Roell ProLine Z005 uniaxial testing device (Zwick/Roell, Ulm, Germany) equipped with a 10 N load cell (Zwick/Roell Xforce HP 10 N) at a strain rate of 0.01 s^-1^. Prior to testing, samples were equilibrium swollen in PBS 1X and carefully measured in thickness and diameter with a calliper (0.05 mm resolution), averaging readings from at least three different points. Force and displacement data were acquired starting with the upper plate of the testing device (connected to the load cell) close to but not in contact with the sample, to guarantee a zero pre-stress initial condition and a constant approach velocity [7,10–13]. Samples were partially immersed in PBS 1X while being tested to preserve their hydration during experiments [7,10–12,14]. Experimental force and displacement data were respectively normalised to sample cross-sectional area and initial length, obtaining engineering stress and strain. Compressive moduli were derived as the slope of the linear portion of the stress-strain plot [15,16].

### Liver organoid generation

Trypsinized cells were re-suspended in liver organoid growth medium (upcyte® Hepatocyte Growth Medium and upcyte® LSEC Growth Medium in 1:1 ratio). 1.0 × 10^6^ upcyte® hepatocytes, 1.0 × 10^6^ upcyte® LSECs and 0.2 × 10^6^ upcyte® MSCs were mixed in 1 mL of liver organoid growth medium, added to the hydrogel-coated plates and incubated (37°C, 5% CO_2_, 95% humidity) for the formation of liver organoids. Cells were cultured under static conditions for up to 72 h, changing the medium at 24, 36, 48 and 60 hours. Culture medium samples from LO-forming substrates collected at different time points after LO formation (i.e. at 24, 36, 48, 60 and 72 h after cell seeding) were stored at -20°C and then biochemically characterised in terms of albumin, urea and glucose content to evaluate LO metabolic activity.

### Biochemical analysis of LO culture medium: Albumin, urea and glucose

Albumin production in 10 μL samples of LO media was measured using an enzyme linked immunosorbent assay (Human Albumin ELISA Quantitation Set, Bethyl laboratories Inc, Montgomery, TX) as per manufacturer’s instructions. Urea secretion was measured using the urease based colorimetric method described in Zawada et al. [17]. Glucose concentration in 10 μL samples was determined by the Yellow Springs Glucose 2300 STAT as per manufacturer’s instructions. Complete liver organoid growth medium (i.e. upcyte® Hepatocyte Growth Medium and upcyte® LSEC Growth Medium in 1:1 ratio) including all the adjuncts (e.g. FBS), was used as a blank for all readings.

### LO oxygen consumption

Oxygen is widely considered as the limiting nutrient for 3D cell cultures [18]. In order to establish the optimal LO culture conditions ensuring long-term organoid viability and functionality, the bulk Oxygen Consumption Rate (*OCR*) of the LO (termed *OCR*_*LO*_) was characterised at specific time-points (i.e. at 24, 48 and 72h after cell seeding).

A commercial needle oxygen sensor (Neofox Phase Measurement system, Ocean Optics Inc, Ostfildern, Germany) was used for O_2_ measurements. A “two point" calibration was performed to establish a linear relationship between the measured signal and the oxygen concentration. Cell culture medium at 37°C in equilibrium with atmospheric oxygen was used as 20.9% O_2_ reference for sensor calibration, while culture medium containing freshly prepared 1% w/v sodium sulphate was used as a reference for 0% oxygen, as per the manufacturer’s instructions. In order to ensure that the LO oxygen consumption is the only cause of oxygen concentration variations over time, the organoid was placed in a modified 1.5 mL Eppendorf tube filled with 1 mL of fresh culture medium at 37°C and, after placing the LO inside, completely sealed to avoid any inward oxygen flux from external atmosphere. Then, the oxygen probe was inserted through the tube at a distance of about 4 mm from the LO. The tube was placed in a water bath at 37°C on an orbital shaker set at 50 rpm, in order to maintain a homogenous (i.e. space-independent) concentration of oxygen within the probed culture medium.

Oxygen concentration was recorded for up to 3h, storing data with a time interval of 5 seconds. Measurements were performed in triplicate at each of the time point investigated. The generic oxygen transport equation to fit experimental data is shown in Eq 1.

$$\frac{\partial c_{O_{2}}}{\partial t}=D\nabla^{2}c_{O_{2}}-u\cdot \nabla c_{O_{2}}+R_{O_{2}}$$ (Eq 1)

where $\frac{\partial c_{O_{2}}}{\partial t}$ denotes the first time derivative of oxygen concentration measured in the culture medium, $D\nabla^{2}c_{O_{2}}$ and $u∙\nabla c_{O_{2}}$ represent oxygen diffusive and convective transport, respectively, and $R_{O_{2}}$ is the LO oxygen consumption. Thanks to the isolation and orbital shaking of the LO-containing Eppendorf tube, no inward oxygen flux is present in the medium and spatial oxygen gradients can be neglected, thus Eq 1 can be reduced to $\frac{\partial c_{O_{2}}}{\partial t}=R_{O_{2}}$. Oxygen consumption is generally modelled with Michaelis-Menten kinetics (i.e. $R_{O_{2}}=\frac{{OCR}_{LO}∙c_{O_{2}}}{K_{m}+c_{O_{2}}}$, with *K*_*m*_ ≈ 7.39 μM for hepatocytes [18–21]), which ensures that at very low oxygen concentrations, where cells barely survive, the oxygen consumption decreases with the available oxygen concentration ($c_{O_{2}}$). To derive the *OCR*_*LO*_ only data where $c_{O_{2}}\gg K_{m}$ were considered (i.e. the first 15 minutes of acquisition), thus oxygen consumption kinetics was approximated as a zero-order reaction (i.e. ${R_{O_{2}}=OCR}_{LO}$) and the *OCR*_*LO*_ derived with a linear fit of experimental oxygen concentration decrease measured over time. Data fitting and analysis were performed using MATLAB (The MathWorks, Inc., Massachusetts USA).

### Modelling LO oxygen transport and consumption

Steady-state multi-physics 3D models which couple oxygen mass transport and consumption to fluid dynamics [18] were developed in COMSOL Multiphysics 3.5a (COMSOL AB, Stockholm, Sweden, 2009) in order to study oxygen concentration profiles inside a liver organoid cultured in a 24-well plate (static culture). Furthermore, given the experimental results on long term LO culture reported in Ramachadran et al. [5], a dynamic system based on the LiveBox1 bioreactor (IVTech srl, Italy), was also investigated. Michaelis-Menten oxygen consumption was considered in the model, imposing *K*_*m*_ = 7.39 μM [18–21] and using the *OCR*_*LO*_ value measured at 24 h, i.e. that immediately after LO formation, representing the worst case.

In particular, each configuration modelled was divided in two sub-domains: i) a fluid domain, in which no oxygen consumption occurs and where the fluid dynamics as well as the oxygen diffusive and convective transport are solved, and ii) the liver organoid, modelled as an ellipsoid (3 mm width, 1 mm height, typical experimental dimensions of the LOs), treated as a solid region where only oxygen diffusion and consumption are solved.

Oxygen transport and consumption in both the static well and the LiveBox1 bioreactor were assumed to be governed by the generic advection and diffusion equation in its non-conservative formulation (i.e., that for an incompressible fluid) [18]. The velocity field resulting from fluid flow convection in the LiveBox1 was solved using the incompressible Navier-Stokes equations for a Newtonian fluid. On the basis of our previous experiments, the inlet velocity in the LiveBox1 was set to *v*_*in*_ = 4.24∙10^−3^ m/s (corresponding to 200 μL/min inflow) [5,22]. Furthermore, since the LiveBox1 bioreactor is realized in PDMS, a gas permeable elastomer [23], the inward oxygen flux through the PDMS bioreactor walls was considered in the model according to the following expression [24]:

$$N_{O_{2},PDMS}=K_{O_{2}}\left(p_{O_{2}}-\frac{c_{O_{2}}}{K_{H,O_{2}}}\right)$$ (Eq 2)

Here, $p_{O_{2}}$ is the ambient oxygen partial pressure (159 mmHg [25]), $K_{H,O_{2}}$ is Henry’s constant for oxygen at 37°C (1.32 × 10^−3^ mol∙m^−3^∙mmHg^−1^ [26]), $c_{O_{2}}$ is the oxygen concentration in the bioreactor culture chamber at the liquid-PDMS interface, $K_{O_{2}}$ is the global mass transfer coefficient, defined as $K_{O_{2}}=P_{m}/L$, where *P*_*m*_ is the oxygen permeability in PDMS (3.786∙10–11 mol∙m∙m^-2^∙s^-1^∙mmHg [23]) and *L* is the PDMS thickness (6 mm in the LiveBox1). To incorporate the inward oxygen flux through the PDMS side walls, the flux boundary condition was applied at the lateral walls of the bioreactor, imposing the oxygen flux of Eq 2. Table 1 summarizes all the boundary conditions imposed in these models.

**Table 1:** Boundary conditions used for the oxygen convection and diffusion and for the Navier-Stokes models.

Model	Surface	Boundary condition
Oxygen convection and diffusion	System side walls	Inward oxygen flux through PDMS ($N_{O_{2},PDMS}$)
	Interface between the LO and the fluid sub-domain	Continuity
	Fluid domain inlet	Constant oxygen concentration ($c_{O_{2}}$ = 0.21 mol/m^3^)
	Fluid domain outlet	Convective flux (***n*** ⋅ (−*D*∇*c*) = 0)
Navier-Stokes	Solid-liquid interfaces	No slip (***u*** = 0)
	Fluid domain inlet	Normal inflow velocity (*v*_*in*_)
	Fluid domain outlet	Pressure, no viscous stress (*p*_0_ = 0)

### Adhesion/cohesion markers

#### Immuno-fluorescence and confocal acquisitions

Adhesion dependent cells, such as hepatocytes express multiple adhesion molecules, including integrins. Of these, Integrin-β1 is crucial for cell attachment to the ECM and for hepatocyte survival and function following trauma [27,28]. Connexin-32 is the major gap junction protein mediating cell-to-cell communication between hepatocytes and underlies a number of liver-specific functions such as glycogenolysis and albumin secretion [29].

In order to investigate whether cell-cell adhesion and cell-substrate cohesion balance is altered between LO-forming and non LO-forming substrates, the expression of adhesive (i.e. Integrin-β1) and cohesive (i.e. Connexin-32) markers was investigated via immunochemistry. All the analyses were performed after 72h of culture. Briefly, LOs were collected and embedded in optimum cutting temperature (OCT) compound for subsequent cryo-sectioning. Slices of 10 μm thickness were obtained with a Leitz 1702 cryostat (Leica GmbH, Bensheim, Germany) and collected on Superfrost^TM^ glass slides (Thermo Scientific). Cells which did not form self-assembled organoids were stained directly on the substrates.

Immunofluorescence was performed as per manufacturer’s instructions using Anti-Integrin beta 1/CD29 rabbit monoclonal antibody (NB110-57123, Novusbio) and Anti-Connexin 32 mouse monoclonal antibody (13–8200, Invitrogen) primary antibodies and Goat Anti-Rabbit IgG H&L (Alexa Fluor 488, ab150077, Abcam) and Goat Anti-Mouse IgG H&L (Alexa Fluor 647, ab150119, Abcam) as respective secondary antibodies. The samples were imaged using a confocal microscope (Nikon A1, Italy). In particular, images were acquired using a 10x objective with a pixel-to-micron ratio of 0.14 μm on a 512x512 matrix. Acquisitions within the green (for Integrin-β1) and red (for Connexin-32) channels were performed using the same confocal settings.

#### Quantitative image analysis

To quantify cell-cell adhesion and cell-substrate cohesion between the different substrates investigated, the Mean Pixel Intensity (MPI) of the objects of interest (i.e. Integrin-β1 and Connexin-32 positive structures respectively on the green and the red channel of each scan) was evaluated using the method detailed in Gonzalez et al. [30]. Briefly, for each channel investigated a global threshold with Otsu’s method [31] was performed to identify the objects. Then, the MPI of the detected objects was calculated in each channel (*ch*) using Eq 3:

$$MPI_{ch}=\frac{\sum_{i=1}^{i=M}I_{M,ch}}{M}$$ (Eq 3)

where M is the number of the object pixels and *I*_*M*,*ch*_ represents the pixel intensity in that channel.

### Statistical analyses

All tests were carried out at least in triplicate. Results are reported as the mean ± standard deviation, unless otherwise noted. Statistical differences were assessed using ANOVA followed by Tukey’s honestly significant difference test (Tukey’s HSD test). The statistical analysis was implemented in OriginPro 9.0 (OriginLab Corporation, Northampton, Massachusetts, USA), setting significance at *p* < 0.05.

## Results

### Biochemical characterisation of liver ECM pre-gel solution

The TCC of ECM pre-gel solution was found to be 19.7 ± 0.2 mg/mL, suggesting that almost all proteins of liver dECM digest (i.e. 20 mg/mL by preparation) are collagenous. The TAC was 213 ± 14 mM. The free amino groups of the liver ECM pre-gel solution allow the use of covalent crosslinking approaches (e.g. chemical, enzymatic) in addition to the physical (i.e. thermal) gelation employed in this work, thus expanding the possible range of applications for this liver-derived material and increasing the tunability of the properties of the hydrogel obtained thereof.

### Mechanical characterisation of agarose and liver ECM gels

**Fig 1:**
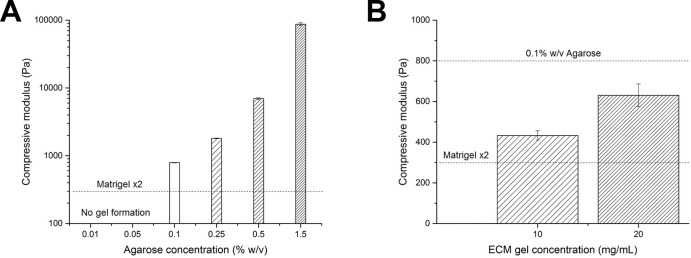
Agarose and liver ECM gels mechanical properties. A) Compressive moduli of agarose gels prepared at different weight/volume (w/v) concentrations. Agarose gels were not formed below 0.1% w/v. The dashed line represents the stiffness of Matrigel-x2 substrates (estimated from the literature). B) Mechanical properties of 20 mg/mL ECM gel and 10 mg/mL ECM-LSEC gel. The two dashed lines represent the stiffness of 0.1% agarose and Matrigel x2 substrates.

The compressive moduli obtained for agarose gels at different concentrations are reported in Fig 1A, showing a non-linear increase of modulus with agarose concentration, as expected [32]. Since no gel formation was obtained at agarose concentrations below 0.1% w/v, this concentration represented the best compromise to mimic Matrigel-LSEC stiffness and hence was chosen as an inert substrate to decouple the effect of mechanical properties from other Matrigel-related signals in triggering liver organoid formation.

The compressive moduli of ECM gels were found to be in between those of 0.1% agarose and Matrigel-LSEC substrates (Fig 1B), with a decrease in compressive modulus with decreasing ECM concentration, as expected [9].

### Liver organoid formation

The matrices used to decouple the effects of substrate stiffness, adjuncts and adhesiveness in kick-starting organoid formation were:

1. Adjunct enriched Matrigel-LSEC (300 Pa) as a positive control for a soft substrate;
2. Adjunct-free Matrigel -PBS (300 Pa) to determine the effects of adjuncts;
3. 1.5% agarose -LSEC (87 kPa) to test for organoid formation on a stiff inert substrate with adjuncts;
4. 1.5% agarose gel (87 KPa) to test for organoid formation on a stiff inert substrate without adjuncts;
5. 0.1% agarose-LSEC (800 Pa) to test for organoid formation on a soft inert substrate with adjuncts;
6. 0.1% agarose gel (800 Pa) to test for organoid formation on a soft inert substrate without adjuncts;
7. 20 mg/mL ECM gel (600 Pa) to test for organoid formation on soft adhesive substrate;
8. 10 mg/mL ECM-PBS gel (400 Pa) to test for organoid formation on a soft adhesive substrate with adjuncts.

Note that the closest Matrigel x2 stiffness matched agarose gel (0.1% w/v) and 1.5% w/v agarose-LSEC gels were investigated to compare results with those obtained by Takebe et al. who observed LO formation on x2 diluted Matrigel with endothelial cell growth medium but no LO formation on 1.5% w/v agarose gels [2].

**Fig 2:**
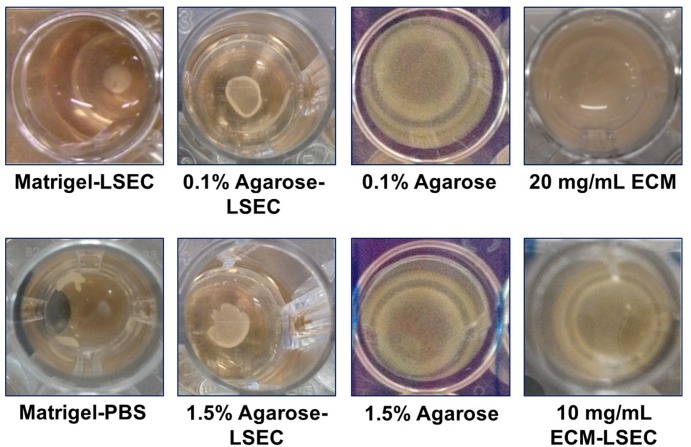
Outcome of liver organoid formation on different substrates at 24 h.

**Fig 3:**
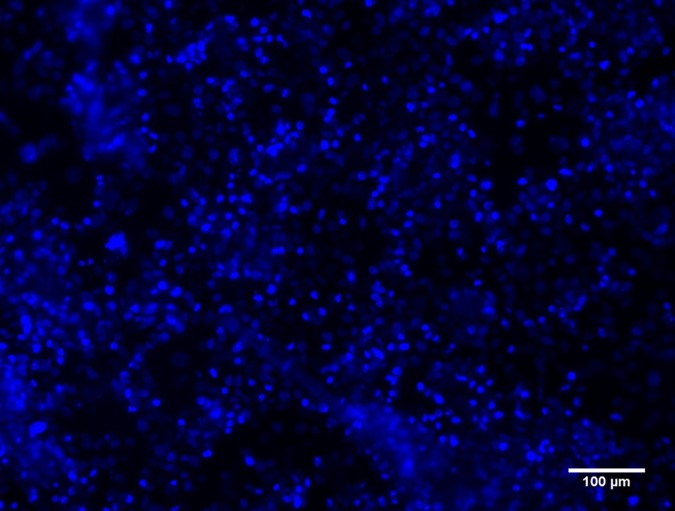
DAPI-stained cells on ECM gel at 24h. A representative image of cells seeded on 20 mg/mL ECM gel is shown.

The outcome of liver organoid formation onto the different substrates at 24 h is shown in Fig 2. When plated on Matrigel-LSEC coated wells, as expected, the three different upcyte® human cell types (hepatocytes, LSECs and MSCs) self-assembled into a compact organoid structure after 24 h, while a poorly stable and partially disrupted LO was formed on Matrigel-PBS. Good LO formation was observed on agarose-LSEC gels both at 0.1% and 1.5% w/v concentration, while no LO formation was observed in the absence of LSEC adjuncts, in agreement with results previously reported by Takebe et al. [2]. As several non-adherent cells were found in the absence of LSEC medium, no further analyses were performed on these agarose substrates. Moreover, neither 20 mg/mL ECM nor 10 mg/mL ECM-LSEC gels were able to support LO assembly. Nuclear staining with DAPI indicated that cells tend to adhere on the ECM gel surfaces rather than forming a LO, likely due to their highly adhesive nature (Fig 3). No differences in cell spreading were observed between cells seeded on 20 mg/mL ECM gels and 10 mg/mL ECM-LSEC gels. Again, these data are in agreement with Takebe’s original report, in which substrates with adjuncts consisting of adhesion ligands (entactin, laminin, collagen), did not support cellular self-assembly [2].

### Biochemical analysis of LO culture medium

**Fig 4:**
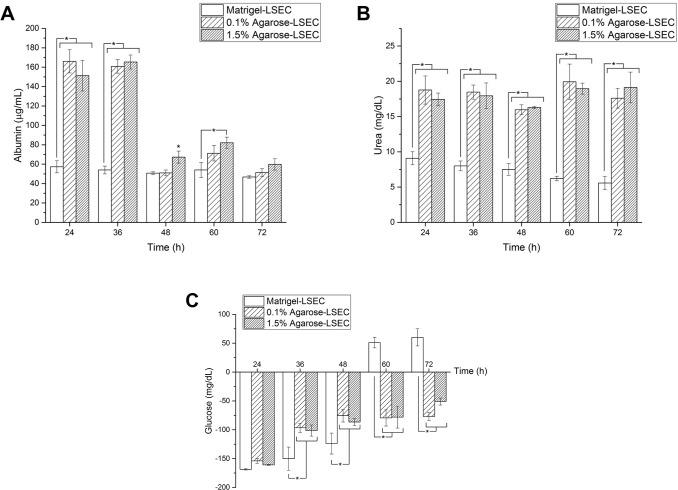
Biochemical analysis of LO culture medium. Albumin (A), urea (B) and glucose (C) production at different time points for liver organoids on different substrates. Significant differences at the same time points (*p*<0.05) are marked with an asterisk.

Metabolic analyses were carried out only for substrates where good LO formation occurred (i.e. Matrigel-LSEC, 0.1% and 1.5% agarose-LSEC). Albumin and urea measured during the static culture are shown in Fig 4A and 4B, respectively. Liver organoids on 0.1% and 1.5% w/v agarose-LSEC gels exhibit a similar trend of albumin and urea production during culture, with no significant differences at the same time point (2-way ANOVA, *p* < 0.05). The only exception is albumin production at 48 h, which is higher on 1.5% agarose-LSEC gels with respect to 0.1% gels. Urea and albumin production of LO on agarose-LSEC gels is generally higher than in LO on Matrigel-LSEC (except for albumin production at 72 h which is equal for all the three substrates investigated).

Fig 4C shows glucose concentrations over time measured during the 72 h static culture. Glucose concentrations measured for LO cultured on 0.1% and 1.5% agarose-LSEC gels exhibit a similar trend, with no significant differences at the same time points. Negative concentrations indicate that liver organoids are consuming glucose from the cell culture medium, while positive values denote glucose production by the liver organoids. In particular, LO cultured on agarose-LSEC gels consumed glucose from cell culture medium until the end of the experiments (i.e. up to 72 h), while those cultured on Matrigel-LSEC consumed glucose until day 2, then started to produce glucose from 60 h onwards. We hypothesise that glucose levels increase on the Matrigel-LSEC compared to the Agarose-LSEC substrates because Matrigel contains growth factors, including EGF (Epidermal Growth Factor) ranging from 0,5 to 1.3 ng/ml. (Suppliers datasheet). As described in the literature [33], EGF can induce the inhibition of the glucose transporter GLUT2. Inhibition of this transporter could result in glycogen degradation and gluconeogenesis [34]. Moreover, cell-matrix interactions could also affect hepatic glucose metabolism, especially in 3D liver organoid systems. In fact, Lu et al. [35] and Wen and coworkers [36] both describe an increase in glucose synthesis in 3D organoid or collagen matrices.

### LO oxygen consumption

**Fig 5:**
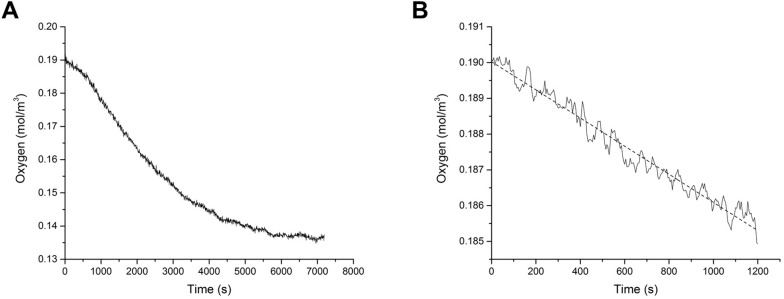
Measurement and analysis of LO oxygen consumption. A) Time-dependent oxygen profile at 24 h after cell seeding. B) Oxygen concentration versus time data measured in the first 15 minutes used to derive the *OCR*_*LO*_ via a linear fit (dashed line).

A typical time-dependent profile of the oxygen concentration is shown in Fig 5A. Only data acquired during the first 15 minutes were considered in the linear fit to derive the *OCR*_*LO*_ (Fig 5B). The *OCR*_*LO*_ at 24 h after cell seeding, i.e. immediately after the LO formation, was equal to 1.39×10^−5^ ± 5.8×10^−7^ mol/m^3^/s, almost 3 times higher with respect to the consumption rates measured at 48 h (4.05×10^−6^ ± 4.9×10^−7^ mol/m^3^/s) and 72 h (4.84×10^−6^ ± 2.62×10^−6^ mol/m^3^/s). This suggests that the organoids suffer from hypoxia during culture under static conditions, as described in [5].

### LO oxygen transport and consumption model

**Fig 6:**
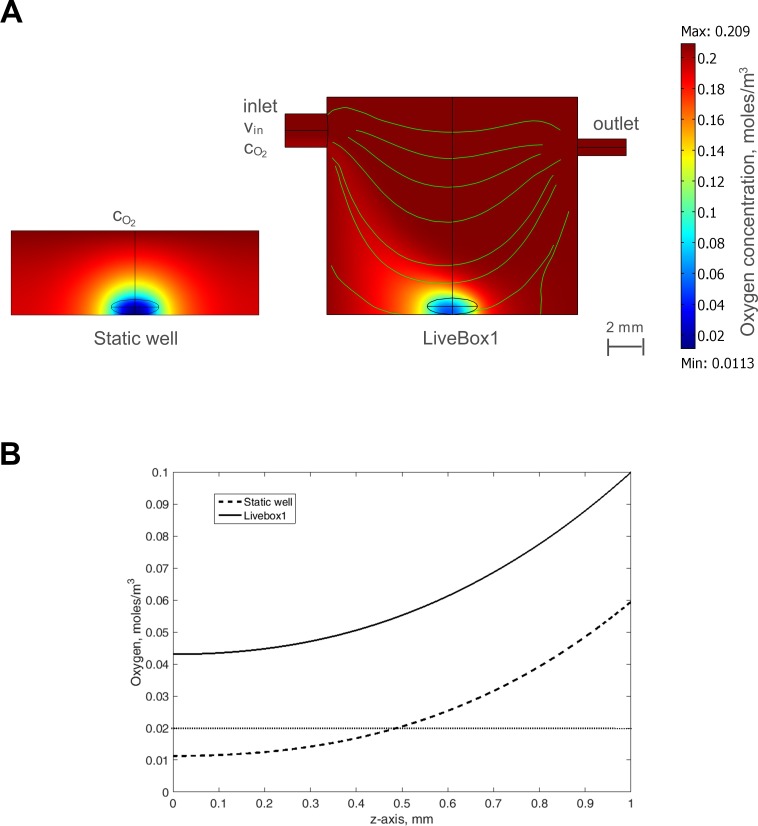
Modelling LO oxygen transport and consumption. A) Results from the computational model, using the measured OCR_LO_ at 24 h. LO in the static well (left) and in the LiveBox1 (right) The computed velocity field in the bioreactor is also shown. B) Oxygen concentration along the vertical axis passing through the centre of the LO (z-axis) cultured either in the LiveBox1 bioreactor (solid line) or in the static well (dashed line). The dotted horizontal line indicates the threshold for hepatocyte function (i.e. 0.02 mM).

Steady-state multi-physics 3D models which couple oxygen mass transport and consumption to fluid dynamics [18] were used to predict oxygen concentration profiles inside a liver organoid during culture in a static well and in a bioreactor. Surface plots showing steady-state oxygen concentrations for the LO cultured in the two configurations are shown in Fig 6A, while oxygen concentration profiles along a vertical line (z-axis) passing through the centre of the LOs are shown in Fig 6B. Under static conditions the oxygen concentration is below the threshold for hepatic function [37] (i.e. 0.02 mM) over a large part of the LO volume, while in the central part of the LO it is below the critical vital concentration (i.e. 0.00264 mM), and could lead to the development of a necrotic core [5]. On the other hand, in the LiveBox1 the oxygen concentration is above the two critical values throughout the LO volume, thanks to fluid flow which enhances the turnover and transport of nutrients. Indeed, the oxygen concentrations remain above the critical values for hepatocytes, confirming that the use of a bioreactor greatly improves LO viability and may allow chronic *in vitro* studies for toxicity or other applications which require long term culture.

### Adhesion/cohesion markers

**Fig 7:**
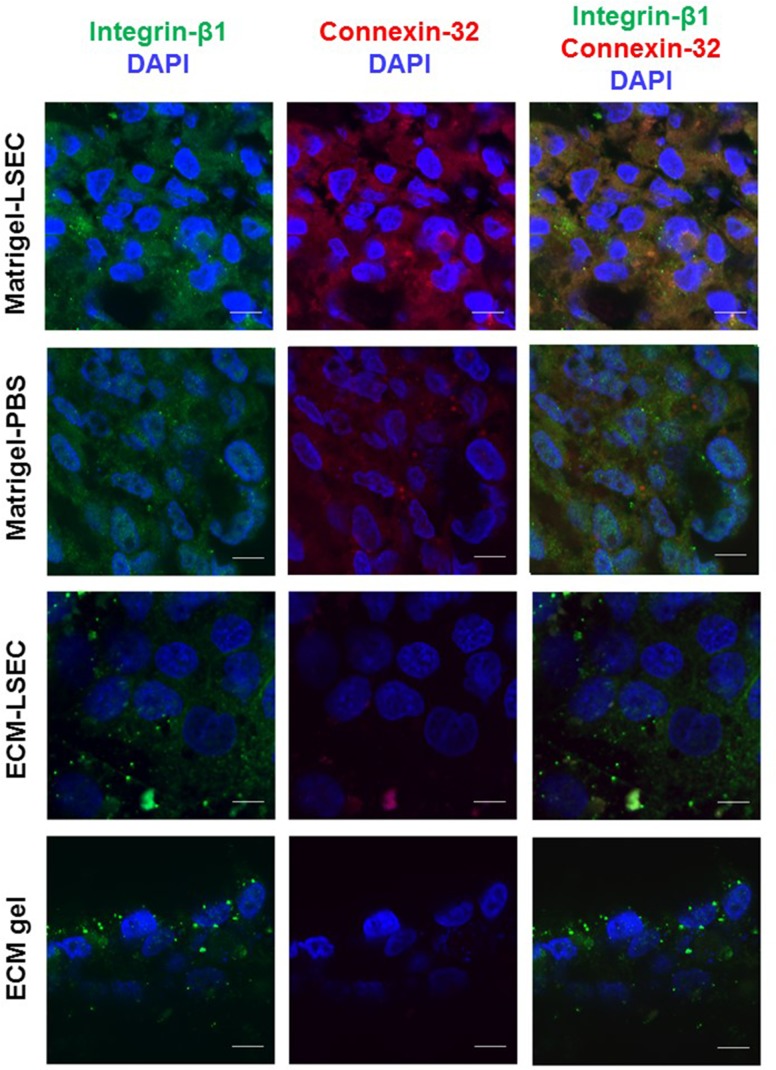
Integrin-ß1 (green, 1^st^ column) and Connexin-32 (red, 2^nd^ column) expression on Matrigel-LSEC, Matrigel-PBS, ECM-LSEC and ECM gel substrates. Blue: cell nuclei (DAPI). Third column: merged image channels. Scale bar: 10 μm.

Liver organoids generated on Matrigel-LSEC substrates showed a level of high expression of both adhesive (Integrin-ß1, green) and cohesive (Connexin-32, red) markers (Fig 7). Lower levels of these two markers were observed for LO obtained on Matrigel-PBS gels, which may explain the poor LO formation. Conversely, cells on non LO-forming substrates, i.e. ECM-LSEC and ECM gels, show a very low expression of adhesive and cohesive markers, with Connexin-32 being almost absent on adjunct-free ECM gels. Notably, cell nuclei were smaller and more rounded in the Matrigel-LSEC organoid, a typical feature of compact 3D tissue morphology [38,39].

**Fig 8:**
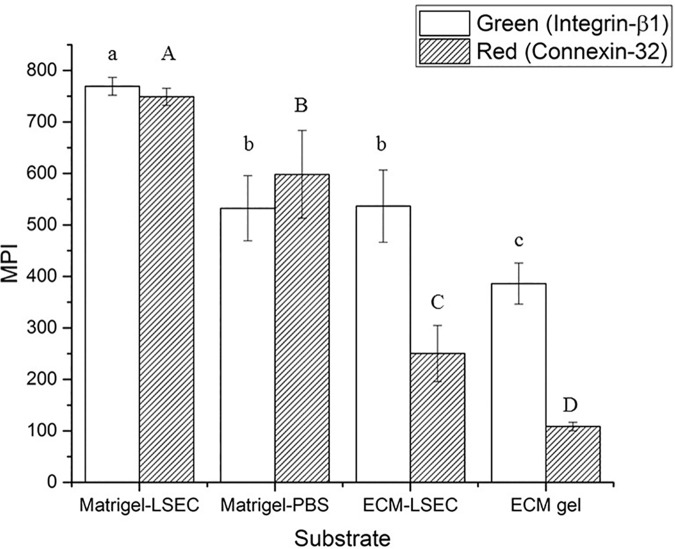
Mean Pixel Intensity (MPI) for green (Integrin-β1) and red (Connexin-32) channels. The MPI was calculated from n = 3 independent experiments per substrate investigated, imaging and analysing n = 3 different DAPI-positive areas in each sample (randomly selected). Different letters indicate significant differences between groups (one-way ANOVA, *p* < 0.05): lowercase letters are referred to ANOVA results of green MPI, while capital letters denote ANOVA results of red MPI. Note that the green and red MPI levels cannot be compared due to differences in the laser power used and in the fluorophore excitation/emission efficiencies.

Quantitative image analyses to determine Mean Pixel Intensities (MPIs) of the two markers confirm the qualitative trends observed. In particular, integrin-ß1 expression decreases significantly from Matrigel-LSEC to both Matrigel-PBS and ECM-LSEC (which showed similar green MPIs), then further decreases in EMC gel (Fig 8). On the other hand, a significant decrease of connexin-32 was found going from Matrigel-LSEC to Matrigel-PBS, ECM-LSEC and ECM gel.

## Discussion

Substrate mechanical properties are known to play a critical role in guiding cell behaviour [7,30], but only one study has investigated how stiffness modulates cell self-assembly into organoids [4]. However, stiffness is only one of the many properties a substrate possesses, and it is likely the balance between stiffness and biochemical factors (e.g. adhesiveness) that promotes the formation of compact cell aggregates. The principal aim of this study was thus to explore LO self-assembly on substrates with different biochemical properties but with similar stiffness.

In 2001 Steinberg and co-workers established that the formation of tissue self-assemblies is essentially a tug-of-war between substrate adhesiveness and cellular cohesion [40]. Our results also suggest that LO formation process is the result of a fine balance between cell-substrate adhesion and cell-cell cohesion. As the ECM gels derive from a decellularised matrix preparation rich with basement membrane proteins (collagen IV, fibronectin and laminin) [8], cell-substrate adhesion is likely to be dominant over cell-cell cohesion on these gels, so cell adhesion is promoted while LO formation is inhibited. This hypothesis was confirmed through the immunofluorescent analyses with specific adhesive and cohesive markers (i.e. Integrin-ß1 and Connexin-32, respectively). In particular, the LO formed on Matrigel-LSEC substrates showed high levels of both Integrin-ß1 and Connexin-32 expression. These two proteins were expressed at lower levels by the poorly stable and partially disrupted LO formed on Matrigel-PBS. Although cells on ECM-LSEC substrates expressed similar levels of Integrin-ß1 as those in the poorly formed LOs obtained on Matrigel-PBS, their expression of Connexin-32 was found to be significantly lower. These results suggest that cell-substrate adhesion (correlated with integrin expression) is likely to be dominant over cell-cell cohesion (related to connexion expression), and may explain the absence of LO formation on ECM-LSEC substrates and confirm the tendency of cells to adhere on the hydrogel surface. The expression of both adhesive and cohesive markers investigated was found to be even lower for cells on ECM gels, with particular reference to connexin-32 which was almost halved with respect to that on ECM-LSEC substrates. As discussed above for the latter substrates, this imbalance between the two markers, in favour of integrin-β1, is coherent with our hypothesis that cells tend to adhere to the substrate surface rather than forming a LO.

Since organoids are of use in a wide number of applications, it is of interest to summarise the substrate dependent factors which contribute to LO formation, using both our data and those reported recently by Takebe et al (Table 2). In the light of the above considerations, an analysis of the table shows that LO formation depends on the biochemical nature of the substrate as well as its stiffness. The two factors interact synergistically to promote (or inhibit) spontaneous LO aggregation. For instance, the ligands provided by the highly diluted (x227) Matrigel coating are sufficient to allow LO formation on relatively stiff (16000 Pa) polyacrylamide substrates, but not on softer (<< 10000 Pa) or stiffer (>> 20000) ones. Even very soft (300 ÷ 1000 Pa) and stiff (>> 20000 Pa) substrates can be used for LO generation, provided the substrate is enriched with some growth factors present in endothelial cell culture media (either the upcyte® LSEC culture medium used in this work or the EGM used in Takebe et al. [2]). However, albeit ECM derived gels are in the same stiffness range as the other very soft substrates investigated, they are likely too rich in adhesion-specific ligands to sustain LO formation. Therefore, as evinced by Table 2, cellular self-assembly is the result of the interaction between the stiffness of a substrate and its biochemical nature. Further studies are necessary to identify precisely which biochemical factors among those contained in Matrigel, commercial growth media and tissue-derived ECM gel play determinant roles in guiding organoid self-aggregation with respect to a given stiffness. In parallel, future investigations on the identification of signal pathways initiated by ECM-specific moieties and/or growth factors related to the induction or repression of integrin and connexin expression should lead to a better understanding of the underlying mechanisms of organoid formation. Nonetheless, our considerations suggest that a window rather than a unique set of substrate properties define the conditions for LO formation.

**Table 2:** Liver organoid formation outcomes on different substrates.

Substrate	Adjunct	Stiffness (Pa)	LO formation	Reference
Matrigel x2	Growth medium	300	✓	2, 5
	None	300	✓ (fragmented)	This paper
Polyacrylamide gel coated with Matrigel x227	None	<< 10000	✕	4
	None	10000–20000	✓	4
	None	>> 20000	✕	4
Agarose 1.5% w/v	None	>> 20000	✕	This paper
	Growth medium	>> 20000	✓	This paper
Agarose 0.1% w/v	None	800	✕	This paper
	Growth medium	800	✓	This paper
ECM gel	None	600	✕	This paper
	Growth medium	400	✕	This paper

In conclusion, we show that liver organoid formation is modulated by the nature of the substrate–specifically by the interplay between stiffness and biochemical ligands–and that the high initial oxygen consumption rate of LOs may lead to hypoxia in the core unless a continuous flux of nutrients is supplied through a fluidic system. The results can be used to design optimal conditions for the generation and culture of LOs *in vitro*.
